# Procedure, Screening, and Cost of Fecal Microbiota Transplantation

**DOI:** 10.7759/cureus.35116

**Published:** 2023-02-17

**Authors:** Austin B Wynn, Garet Beyer, Megan Richards, Lisa A Ennis

**Affiliations:** 1 Public Health, Environmental Health, Informatics, Information Sciences, Alabama College of Osteopathic Medicine, Dothan, USA

**Keywords:** screening protocol, fecal microbiota, cost efficacy, procedure safety, fecal microbiota transplantation (fmt)

## Abstract

Fecal microbiota transplantation (FMT) is currently considered a potential treatment for various GI-related illnesses, with the goal to replenish natural healthy flora of the GI tract that has been harmed because of antibiotic use or overgrowth of harmful bacteria. Current methods of administering the processed stool include colonoscopy and enema, while an oral capsule is being developed. Each method of administration carries its own set of risks, including adverse reactions to treatment, infection following the invasive administration procedure, and flare-ups of GI-related symptoms. Current oral administration through nasoduodenal tube poses a risk for aspiration which has not been ruled out as the cause of subsequent pneumonia and death in patient trials. The development of an oral capsule could address some of the faults of the current methods, not only making treatment more affordable and accessible but also less of a risk due to its minimally invasive nature. FMT is also a treatment option to attenuate adverse effects associated with antibiotic use, including combatting the emergence of antibiotic resistance, as well as adverse effects related to other medical treatments such as chemotherapy. While FMT is an unexplored treatment option for multiple gastrointestinal disorders and is currently still largely inaccessible for many patients financially, studies have suggested that it could be a more affordable treatment option long-term for patients as aspects of the treatment become more affordable with further research.

## Introduction and background

Fecal microbiota transplantation (FMT) is the process of taking feces from a healthy donor, a volunteer to donate fecal material and have it screened for multiple types of bacteria for a combination that passes basic criteria from the Food and Drug Administration (FDA) and any additional criteria given by specific studies, and implanting it into the gastrointestinal tract of a patient suffering from a GI-related illness. FMT is a focused treatment to replenish the natural flora of the GI tract to overcome the overgrowth of harmful bacteria. The process involves mixing the donated stool with a bacteriostatic liquid, a liquid that prevents bacterial reproduction, and removal of particulate matter from the sample. The processed stool is then administered to the patient. Colonoscopy and enema are the two major administration processes and oral capsules are currently in development as an easier administration procedure. These capsules would allow for outpatient delivery of the treatment.

It is important that patients receiving FMT stop taking antibiotics a day or two before administration to preserve the implanted microbiota [1]. There are multiple routes of administration of the stool samples. Oral administration, through a pill containing freeze-dried fecal matter, provides no procedure-related risk like other routes and is a cost-effective treatment option. Although this is an easier way of administering FMT, more studies are needed to determine if efficacy is significantly different than other routes. The current oral administration is through nasogastric tubing. This route has a risk of aspiration and discomfort due to tube placement for the patient. Delivery via colonoscopy allows for evaluation of the mucosa and seems to be the most effective administration route for *Clostridioides difficile* infections (CDI) given the location of administration [1]. Despite its effectiveness, this route is invasive, expensive, and is associated with general colonoscopy risks like sedation risk and bleeding due to perforation of the GI tract. Enemas, a procedure in which a liquid or a gas is injected into the rectum and then expelled, can be a less invasive method compared to colonoscopy and require no sedation and have a lower cost. FMT delivered through an enema can cause issues like the treatment reaching the right side of the colon making it less effective for CDI treatment [1]. A new method of delivery being studied is the administration of capsules filled with freeze-dried donor samples, or lyophilized stool, that can be taken at regular intervals in clinical or non-clinical settings. Frozen samples have been under investigation for their effectiveness in comparison to fresh and freeze-dried stool samples and revealed similar outcomes, therefore making encapsulated administration a prime method for treating intestinal disorders at specific locations [2]. A problem in oral administration is the site the pills target. These locations are effective at disorders higher in the intestinal tract, but further studies are needed to determine the reach of oral administration. Storage of the freeze-dried sample can last up to six months and studies at 10-month storage show little change in the effectiveness [2]. This would allow for long-term storage for re-administration of pre-donated, healthy samples derived from the patient during their healthy state. Although the storage and relative cost of FMT via frozen or freeze-dried samples could be beneficial for patient care, the current screening for donor samples is unregulated and excessively exclusive.

Screening of the patient and donor for FMT is vital to the sample's successful administration. Strict exclusion criteria for donor samples are present regardless of the reviewed studies, but criteria differ with each study due to the irregular guidelines by the FDA [3]. Each criterion includes a stool and serum blood analysis and a clinical and social risk assessment for possible gut-brain axis transmittable factors. Any type of antibiotic use will exclude patients from treatment with FMT, as will the presence of any form of GI disorder [1]. Many studies have been done on the significance of the donor’s relationship with the patient. Some investigators hypothesize a complete reset of the microbiome with anonymous donors allows for a better outcome, although more randomized studies need to demonstrate this hypothesis [1]. An additional option to screening is autologous donation. This donation type is from the patient themselves during a remission of disease pathology and reused during relapse of symptoms [2]. Stool banks have made the process of donor selection more accessible to the general population. With the increase in stool banks and FMT potentially becoming more popular, accessibility to treatment will be further increased, allowing for effectiveness in large populations. More testing and consistent guidelines are still needed to capitalize increases in fecal sample donation and the longevity of samples taken.

## Review

Fecal transplants and antibiotic side-effects

Antibiotics have many beneficial qualities but have limitations that include the potential for the development of microbial resistance to the drug. Fecal transplants can help with adverse effects associated with the use of antibiotics, including the development of antibiotic resistance, and can help with adverse GI effects associated with chemotherapy. Commensal bacteria in the gut microbiome are useful in the control and removal of harmful bacteria. Antibiotics affect the natural microbiome of the intestinal tract by targeting all bacterium types. Fecal transplants could be a treatment option to add commensal bacteria back to the gut after dysbiosis occurs. A few studies demonstrated the effectiveness of FMT for use in gut microbiome reorganization. FMT has been used to assist in treating allo-hematopoietic stem cell transplant patients, who are also taking antibiotics, to use alongside antibiotics to maintain a diverse gut microbiome rather than concomitant use of probiotics with antibiotics, and for the treatment and cure for *C. difficile* along with the use of vancomycin, by reconstructing the microbiome to grow bacteria that are beneficial to the individual [3-9]. From the reviewed studies, the primary focus of using FMT is to maintain a gut microbiome capable of handling extreme changes, or reconstructing an already unbalanced biome to one that is synergistic to the host. Probiotics have a similar concept by providing specific bacteria to the host, but results suggest that probiotic use after treatment with antibiotics increased specific species >100 fold and returned to baseline once probiotic use was stopped. Probiotics also delayed the reconstitution of the indigenous microbiome. FMT gave comparable results to the mouse study and returned microbiome diversity to baseline within one day of the transplant [9]. Although probiotics could be effective in maintaining a balanced microbiome in healthy individuals, the studies reviewed did not ensure effective use in sick individuals without continuous use. A common illness affecting the gut microbiome is *C. difficile*. It can easily upset a balanced environment and create a location for ‘bad’ bacteria to continue growth, allowing for recurrent infections. The best way to fight these infections is strong antibiotic use, and although effective, they create a biome that allows for reinfection. Studies have shown that the use of FMT alongside antibiotic use in *C. difficile* patients allows for the microbiome to be reconstructed to a healthy balance and provide colonization resistance, preventing reinfections from occurring [5-7]. Better microenvironments can lead to better health outcomes and decrease mortality rates and lead to more cured patients [8]. With more cured patients the first time treating and limiting the spread of *C. difficile* in hospital settings, the cost of care could decrease significantly while increasing health outcomes and decreasing mortality.

The invasive techniques of fecal transplants

Fecal microbiota transplants have the potential to cause infection given the bacterial diversity of stool samples. Risks associated with FMT are also increased due to invasive procedures performed to transplant the donor samples into the GI tract of the patient. A study in the Journal of United European Gastroenterology treated 39 patients with FMT [10]. Of the 39 patients, 32 were in remission of *C. difficile* six months later. Although these results show promise for FMT as a viable treatment option for *C. difficile*, serious adverse effects did occur. Nine of the patients developed adverse effects 12 weeks post-FMT, five of which were related to the FMT procedure. One patient died 15 days after the FMT due to pneumonia despite antibiotic treatment. A causal relation to FMT could not be excluded. Per the study, the donor sample was administered through a nasoduodenal tube and regurgitation was observed afterward. The authors of this study suggest the regurgitation could be responsible for aspiration of donor feces which could have led to pneumonia. Within the other four patients, adverse effects like nausea and diarrhea seemed to be the fundamental issues following the procedure [10].

A review to assess the safety of FMT procedures found flares of irritable bowel disease in 14.3% of cases post-FMT [11]. The amount of post-FMT inflammatory bowel disease (IBD) issues were observed through a meta-analysis of IBD cases treated with FMT. When comparing the delivery method of FMT to IBD flares, which is the recurrence of symptoms after remission, delivery through the lower GI tract caused more flares than nasogastric tube administration [11]. Issues in medical procedures, specifically in surgical procedures, tend to have a higher risk of infection owing to the increased invasiveness of these procedures. Fecal transplantation via colonoscopy or other surgical procedures will have a higher risk of complications although they are effective methods of administration. To prevent the complications of invasive procedures, an oral pill is in development that will have similar effectiveness but reduced risk. A randomized study comparing capsule use and colonoscopy used in fecal transplants saw prevention of recurrent *C. difficile* in 96.2% of patients from each group. Minor adverse effects were 5.4% for capsule use as opposed to 12.5% in colonoscopy procedures [12]. The oral capsule shows nearly identical results for use of fecal material in the treatment of dysbiosis. This study provides evidence that fecal transplants are currently being developed into a safer method of administration, while maintaining the goal of becoming an effective method for the treatment of gastrointestinal diseases. More recent evidence has compared differing FMT treatment options and compared their effectiveness in curing antibiotic-associated destruction of the gut microbiome. In this study, human fecal material was prepped for fecal transplantation using differing techniques dependent on the type of FMT transplant used. Mouse models were used and gut microbiome, including diversity studies and chemical content studies, were performed before antibiotic treatment, leading up to FMT use, and 10 days after FMT use. This study found that all FMT treatments were successful in creating a stable microbiome within a 10-day period. They also found that the lowest enema dose was equally efficient as the highest enema dose given. More excitingly, the oral capsule given was slightly less efficient than enema dosing although the oral capsule contained 100 times less bacteria and was given for five more days than the enema. The oral capsule was also equally effective when frozen or lyophilized, which creates more options for decreasing cost since freezing capsules is easier and cheaper than lyophilization of the contents [13]. This study opens the door for more affordable treatment options for FMT use and may aid in the decreased cost of such treatments.

The economic impact of FMT

Fecal transplants are viewed as an expensive process due to the surgical nature of most FMT procedures. In addition, excessively strict inclusion criteria and the lack of potential donors also contribute to increased costs by limiting the number of healthy donor fecal matter available for transfer. Due to this, fecal transplantation procedures can be costly and antibiotic treatments offer an affordable option. To assess this issue, one multicenter trial aimed to evaluate the increasing demand for donor samples. Many facilities allow patients to choose a donor, which may not always be possible. Initially, the Food and Drug Administration called for a mandatory shutdown of all research into FMT until a full Investigational New Drug approval process was made [14]. More research can now be done on the effectiveness of FMT on multiple disorders, although the donor samples are still difficult to collect. Many FMT procedures are also covered by insurance due to the procedure taking place during a colonoscopy for CDI patients.

In one study, 116 potential donors were prescreened using the FMT Working Group guidelines with minor changes to not look for *H. pylori* antigens and they performed extensive blood testing to look for undiagnosed pathology [15]. Only 12 donors were enrolled in the study after exclusions were made. A substantial portion of the potential donors failed stool and blood prescreening, and the rest of the potential donors who did not get enrolled refused long-term commitment to the program [15]. Donor criterion includes much more than consideration of potentially harmful bacteria present in donor samples. Protozoa are also important in fecal screenings due to their presence in healthy individuals. Screening is also done for obesity and metabolic-related diseases. Notable metabolic changes have occurred in patients after FMT due to specific metabolites present in donor samples [16]. Finding donors is difficult, but a further increase in studies and knowledge will aid in the understanding of the process leading to improved recruitment of potential donors.

In a recent review, the cost of fecal transplants was found to be lower in comparison to antibiotic treatment [17]. Different fecal transplant procedures were compared and showed that they are lower in cost than current antibiotic treatments used in clinical practice and could limit the amount of time spent in hospitals. Furthermore, FMT treatments can now be given orally, and this will lower the cost by avoiding expensive and dangerous invasive procedures [17]. FMT is an unexplored treatment option that has potential problems that need to be better understood, but although problems exist with FMT, recent studies suggest it is the most affordable and effective treatment option for multiple gastrointestinal disorders.

Recent studies have looked at the cost-effectiveness of FMT when compared to antibiotic drugs for the treatment of hospital-acquired *C. difficile* infection (CDI). One study in Canada used a Markov model to simulate a 65-year-old patient with second recurrence of mild-to-moderate CDI. They compared treatment options in areas with FMT programs and without an established program. After analysis and conversion to US dollars, outpatient vancomycin was $419.05 per course of treatment and fidaxomicin, brand name Dificid, was $1552.88 per course of treatment. FMT via capsule was $2097.39 [18]. Vancomycin and fidaxomicin are common first-line therapies for CDI and have a high potential for recurrence, which is shown in the study for probability of success in curing CDI. Vancomycin had a success rate of 0.556 and fidaxomicin had a success rate of 0.710, while FMT’s lowest success rate was 0.898 with capsule transmission of the healthy gut bacteria [18]. The total cost of FMT, including all aspects of treatment like screening and prep of the sample, was estimated at $3510.26 when compared to the use and prep of vancomycin and $3422.43 when compared to the use and prep of fidaxomicin. This cost is for FMT via colonoscopy, which was the highest cost estimated for FMT, and the number to treat to be comparable to antibiotics was 15 and 16, respectively. These costs seem to be more than what is currently available for patients and less cost-effective overall. One aspect to consider when comparing cost is hospitalization for patients with recurring CDI, which is a common occurrence with CDI after treatment with antibiotics. Hospitalization cost was checked during the study mentioned above. The cost for hospitalization due to mild-to-moderate recurrent CDI was $2688.94, $5252.99 for severe CDI that does not require a colectomy, and $17,082.18 for severe CDI that did require a colectomy [18]. Another U.S. study performed a similar study using a Markov model of a 67-year-old patient and found that success rates of vancomycin and fidaxomicin were higher at 0.846 and 0.800 for severe infections, respectively [19]. FMT may currently have higher costs that can be lowered over time, like screenings that are at a current cost of $883.60, but treatment of patients with pharmaceuticals that have less than 75% success rates may lead to worsening patient outcomes, increased mortality rates, and overall higher cost due to hospitalization of these patients [19].

## Conclusions

Current studies show that FMT can serve as an effective treatment option for some GI-related illnesses when given in short timeframes. Promising evidence of effectiveness and longevity of remission after treatment when compared to both controls and probiotic treatment groups has been seen, but it is still largely inaccessible. This is due to several factors, including the invasiveness of current delivery methods, financial impacts, and the limited availability of donor samples. The highly exclusionary criteria for donors as well as the current limited knowledge on the collection and longevity of samples not only limit the accessibility of FMT as a treatment option but also limit the rate that new research on this subject can be completed.

Overall, there is much room for further research on many aspects of FMT, including improving the overall efficiency and accessibility of collecting donor samples and minimizing the risk associated with both current methods of delivery as well recurrence of illness following treatment. FMT screening before donation could be adjusted to agreed-upon criteria in the medical community in order to regulate the process. With more donor samples, further studies could determine the effect types of bacteria needed and increase efficacy of FMT treatment. Along with increased efficacy, changes to route of administration could decrease adverse drug reactions and allow for better drug profiles. Future research into these areas should improve the safety of FMT and make it a more financially accessible treatment option for patients.
